# Use of Glucocorticoids in SLE: A Clinical Approach

**DOI:** 10.31138/mjr.230124.uos

**Published:** 2024-06-30

**Authors:** Daniel Martin-Iglesias, Diana Paredes-Ruiz, Guillermo Ruiz-Irastorza

**Affiliations:** 1 Autoimmune Diseases Research Unit, Biobizkaia Health Research Institute, Department of Internal Medicine, Hospital Universitario Cruces, Spain,; 2 University of the Basque Country, Bizkaia, the Basque Country, Spain

**Keywords:** glucocorticoids, methylprednisolone, prednisone, remission, lupus activity, lupus nephritis, toxicity, damage

## Abstract

Glucocorticoids (GCs) are one of the most effective first-line treatments for systemic lupus erythematosus (SLE). However, GC burden is associated with damage. The initial GC dose and tapering schedule should be tailored to the severity of the clinical scenario. As lupus therapy should prompt remission while minimising damage, recent guidelines recommend a more accurate approach to the use of GCs, setting lower starting doses and rapid tapering schemes, and encouraging maintenance prednisolone doses <5 mg/day. Methylprednisolone pulses (MP) help to reduce the dose of oral GCs and improve the clinical response in both severe and non-severe manifestations, without significant side effects. Fixed-tapering GC scheme provides a useful strategy to reduce GCs exposure. Long-term antimalarial treatment and early initiation of immunosuppressive drugs improve clinical efficacy while reducing GC toxicity. Besides, withdrawal of GCs is an achievable goal in patients in prolonged remission on stable treatment, and recent studies have attempted to identify the most suitable candidates. In this article, we review the pharmacological basis, clinical evidence of efficacy, dose-related harms, and potential withdrawal of GCs. We also review guidelines recommendations and finally give a personal and practical approach to dealing with the use of GCs in SLE patients.

## INTRODUCTION

Activity and damage are among the main prognostic predictors of systemic lupus erythematosus (SLE). Therefore, the main goals of lupus management are to achieve rapid remission, avoid flares and prevent damage.^1^ The international task force on definitions of remission in SLE (DORIS) has proposed a definition, based on clinical systemic lupus erythematosus disease activity index (SLEDAI)=0, Physician’s Global Assessment (PGA) <0.5 (0–3), prednisolone <5 mg/day, and stable treatment with antimalarials, immunosuppressive drugs, and biologics.^2^ In addition, the Asia-Pacific Lupus Collaboration group has proposed the concept of low disease activity state (LLDAS), based on SLEDAI ≤4, no new disease activity, PGA ≤1, prednisone ≤7.5 mg/day, and absence of adverse events of immunosuppressive drugs or biologics.^3^ Treatment should aim to achieve remission, as defined by the DORIS criteria, or at least LLDAS.^1^ Glucocorticoids (GCs) are considered one of the most effective first-line treatments for SLE. However, their known toxicity at medium-high doses makes them a double-edged sword. The purpose of this review is to update the current evidence on the management of GCs in SLE patients from a practical perspective.

## HISTORICAL TRENDS IN THE USE OF GCs IN SLE

Since the first clinical use of the miraculous E compound by Hench in 1948, GCs have been considered one of the cornerstones of lupus therapy.^4^ However, high daily and cumulative doses of oral GCs are known to be associated with damage.^4–7^ Clinical guidelines^1,8–14^ (see **Table 1**) have traditionally not provided clinicians with detailed schemes on the use of GC, arguing the lack of clinical trials, despite the on-growing observational data exploring the most effective doses or regimens of administration^5,15–18^ Classic recommendations, starting with the 2012 ACR guidelines for lupus nephritis,^8^ have been based on three main principles: 1) high initial doses of GC, usually 1 mg/kg/day (an indication based more on custom than on evidence^19^); 2) use of intravenous methylprednisolone pulses (MP) only for severe or life-threatening conditions; and 3) maintenance GC doses under the rather vague umbrella of “the lowest possible dose”. Although there is no universal agreement regarding the definition of low-dose GCs, the cut-off of ≤7.5 mg/day of prednisone or equivalent has been generally accepted,^19^ however, doses ≤5 mg/day are probably more appropriate and already recommended in the most recent guidelines.^1^ Over the last decades, there were no detailed tapering schemes and daily GC dose was supposed to be driven by patients’ clinical course and physician preferences. This approach gave the use of GC an aura of being “more an art than a science”. Besides, such undefinition led patients to be exposed to high burdens of GCs, with the implications for the risk of damage accrual. Fortunately, more recent guidelines^9,11^ have improved in offering clinicians a more practical approach for GC use. One of the major improvements was to set up the initial prednisone doses below the “classic 1 mg/kg/day”, usually at 0.5 mg/kg/day or 30 mg/day maximum. This initial dose, with rapid tapering, would translate into a much lower GC load. In addition, guidelines^9^ have started to expand the indication of MP beyond life-threatening conditions, widening the recommendations for using pulses also in moderate-severe SLE activity, which should be followed by prescribing reduced doses and more rapid tapering of oral prednisone. Indeed, the KDIGO guidelines for lupus nephritis (LN)^14^ include a detailed tapering scheme targeting the dose of 5 mg/day in 11 weeks, almost matching our own approach since 2009 in the Lupus-Cruces LN protocol.^18,20,21^ All these constitute important milestones which would finally lead the myth of the GC use in SLE as a form of art to come to an end.

**Table 1.** Recommendations for the use of glucocorticoids in Systemic Lupus Erythematosus practice guidelines.

**Guideline (year) [ref]**	**Clinical setting**	**Pulse therapy**	**Initial dose of prednisone**	**Tapering scheme**	**Maintenance dose**	**Discontinuation scheme**
**ACR (2012)** ^8^	LN III–IV	500–1000 mg/day methylprednisolone for 1–3 days	0.5–1 mg/kg/d	Not specified. “For a few weeks”	Not specified. “To lowest effective dose”	Not specified
	LN V	NO	Prednisone 0.5 mg/kg/d	Not specified. Maintain initial dose by for 6 months	Not specified.	Not specified
**BSR (2018)** ^9^	Mild activity	Not indicated	≤20 mg/day	Not specified. Maintain initial dose for 4 weeks. Taper over several weeks	≤7.5 mg/day	Immunosuppression for at least 3 years, in combination with low dose prednisone. Gradual drug withdrawal, GCs first.
	Moderate activity	MP ≤250 mg/day for 1–3 days	≤0.5 mg/kg/day if no MP	Not specified		
	Severe activity	MP 500–750 mg/day for 1–3 d Severe activity ays	≤0.75–1 mg/kg/day if no MP OR ≤0.5 mg/kg/day + MP	Not specified			
**GLADEL/PANLAR (2019)** ^10^	LN	Not indicated	1–2 mg/kg/day, maximum 60 mg/day for paediatric patients. No scheme proposed for adult patients	Not specified Lowest doses for the shortest period	≤7.5 mg/day	Not specified
	Diffuse alveolar haemorrhage	Indicated, no specific scheme proposed	Not specified			
**EULAR (2019)** ^11^	Mild-moderate flare	Not indicated	≤0.5 mg/kg/d	Not specified Gradual tapering recommended	≤7.5 mg/d	Not specified Discontinue when possible Prompt initiation of immunomodulatory agents can expedite the discontinuation of GC
	Severe/organ-threatening disease:	Consider MP 250–1000 mg/d for 1–3 days	0.5–0.7 mg/kg/d	Not specified Gradual tapering recommended			
**EULAR/ERA-EDTA (2020)** ^12^	LN III-IV	MP total dose 500–2500 mg/day, depending on disease severity	Prednisone 0.3–0.5 mg/kg/day	Prednisone 0.3–0.5 mg/kg/day for up to 4 weeks, tapered to ≤7.5 mg/day by 3 to 6 months	≤7.5 mg/day	Not specified. Gradual withdrawal of treatment (GC first, then IS), when at least 3–5 years in complete clinical response.
	LN V		Prednisone 20 mg/day	Prednisone tapered to ≤5 mg/day by 3 months	≤5 mg/day	
**KDIGO (2021)** ^13^	LN III–IV	MP 250–500 mg/day for 3 days	Prednisone 0.8–1 mg/kg/day OR Prednisone 0.6–0.7 mg/kg/day OR Prednisone 0.5–0.6 mg/kg/day (max 40 mg/day)	Prednisone tapered to 5 mg/day at week 21 OR Prednisone tapered to 5 mg/day at week 17 OR Prednisone tapered to 5 mg/day at week 11	≤7.5 mg/day	Not specified. May be considered after complete clinical response for about a year and with no extrarenal disease.
	LN V	No specified GCs scheme				
**EULAR (2023)** ^1^	Mild-moderate flare	Not indicated	≤0.5 mg/kg/day	Not specified Gradual tapering recommended	≤5 mg/day	Not specified. Discontinue “when possible”. Prompt initiation of immunomodulatory agents. can expedite the discontinuation of GCs.
	Severe/organ-threatening disease	Consider MP 250–1000 mg/day for 1–3 days	0.5–0.7 mg/kg/day	Not specified Gradual tapering recommended			
**KDIGO (2024)** ^14^	LN class III-IV-V	MP 250–500 mg/day for 3 days	0.5–0.6 mg/kg (max 40 mg)	Tapering scheme detailed in week-by-week bases reaching 2.5 mg/day by week 13	Low-dose prednisone (2.5 mg/day)	Consider GC complete withdrawal after at least 12 months after clinical remission

LN: Lupus nephritis; SLE: systemic lupus erythematosus; MP: methyl-prednisolone pulses; HCQ: hydroxychloroquine; GCs: glucocorticoids; CNS: central nervous system.

## GENOMIC AND NON-GENOMIC PATHS: THE CLUE OF CLINICAL EFFICACY AND DAMAGE OF GCs

GCs work through genomic and non-genomic routes. Both differ in the underlying molecular mechanisms and in the balance between beneficial and adverse effects.^22,23^ The genomic pathway is initiated by the binding of GC to the cytosolic-GC receptor (cGR). The resulting transrepression leads to reduced synthesis of proinflammatory cytokines.^24,25^ On the other hand, transactivation is responsible for most of the adverse metabolic effects of GC, such as insulin resistance, skin atrophy and bone resorption.^26^ Both simultaneous mechanisms explain why GC-related toxicity increases in parallel with the anti-inflammatory activity as the genomic pathway becomes more activated.^26–29^

In contrast, the non-genomic pathway relies on three main mechanisms, independent from the binomial transrepression/transactivation, which are mediated either by the GC-cGR complex, by membrane-bound GR (mGR) or by non-specific interactions with cellular membranes. Non-genomic mechanisms result in the modulation of immune cells with a rapid onset of action and almost null metabolic adverse effects. In addition, the activation of mGR can also influence gene expression, thereby enhancing anti-inflammatory genomic effects.^26–31^

The activation of the genomic and non-genomic pathways is different for the different doses of GC. Up to 7.5 mg/day of prednisone or equivalent, the saturation of the genomic pathway cGRs is less than 50%. Up to 30 mg/day, the cGRs are progressively saturated above 50%, reaching almost complete saturation at doses between 30 and 100 mg/day. In contrast, the activation of the non-genomic pathway starts at prednisone-equivalent doses above 100 mg/day^28^ and maximum immunomodulatory actions are reached at doses between 250 and 500 mg/day.^32,33^ This means that 30 mg/day is the threshold for maximum both beneficial and toxic effects of the genomic pathway, and doses of 250 mg/day result in almost maximum clinical effects mediated by the non-genomic pathway. Dexamethasone and methyl-prednisolone are far more potent than prednisone in activating non-genomic mechanisms and thus the preferred GCs for pulse therapy.^34^

## THE THERAPEUTIC VALUE OF GCs

GCs are among the most potent anti-inflammatory drugs and the first-line initial and maintenance therapy for the inflammatory manifestations of SLE.^1,10^ In terms of efficacy, no clinical trial has ever compared the effects of higher and lower doses of prednisone in general lupus patients. Nevertheless, randomised controlled trials and observational studies in LN provide sufficient data on the efficacy of different prednisone doses in the prototypical severe form of SLE.^19^ Response rates do not seem different between those studies using high initial doses of 1 mg/kg/day of prednisone and those combining MP with lower doses between 0.3 and 0.5 mg/kg/day, whether cyclophosphamide or mycophenolate were used.^35–40^ In addition, a major role for MP in the achievement of a clinical response has been shown in lupus patients with severe disease manifestations. Observational studies from the Lupus Cruces-Bordeaux cohorts have addressed the efficacy of the Lupus-Cruces LN protocol, which consists of induction therapy with starting doses of prednisone 20–30 mg/day combined with 125 mg MP with each fortnightly pulse of 500 mg of cyclophosphamide.^17,18,21^ The previous studies show that repeated pulses of MP help to reduce the dose of oral GCs and improve clinical response.

Induction therapy must be always adjusted to the severity of the clinical scenarios.^4^ Doses of prednisone not higher than 7.5 mg/day, 15 mg/day or 30 mg/day should be enough to treat mild, moderate or severe flares, respectively.^4^ Doses of prednisone higher than 30 mg/day increase toxicity without significant additional therapeutic effects. The administration of MP, by activating the non-genomic mechanisms, generate a rapid and potent anti-inflammatory effect in moderate to severe flares of SLE and can be also considered for mild flares unresponsive to prednisone up to 7.5 to 10 mg/day within one week. After remission, maintenance therapy with doses of prednisone ≤5 mg/day should be continued to prevent future flares.^1,10^

## GCs DOSE-DEPENDENT ADVERSE EFFECTS

High doses of GCs are associated with an increased incidence of infections^41^ and irreversible organ damage, mainly cardiovascular events, osteoporotic fractures and osteonecrosis.^4–7^ The contribution of GC to damage in SLE patients has been shown to be lower in the early course of the disease, but GCs account for most of the damage accrued after prolonged exposure.^42^ The burden of prednisone during the first year is mainly dependent on the dose given during the first month.^43^ A recent study using receiver operating characteristic (ROC) curves has suggested that the cut-off points of prednisone dose related to damage at 5 years was over 30 mg/day for the first month and over 7.4 mg/day for the first year of treatment.^44^ Moreover, it has been consistently shown that prednisone <7.5 mg/day and pulse therapy are not associated with the increased rate of infections and damage accrual seen with conventional medium-high dose oral GCs schemes.^5,7,32,33^

### Infections

SLE patients have a significantly increased risk of developing infections. Although patients with high baseline activity have a higher risk of infection in the first few months, GCs play the main role. In the Lupus-Cruces and RELES cohorts, treatment with medium to high doses of prednisone was the main predictor of major infectious events.^41,45^ In contrast, antimalarials had a protective effect in both studies. Results from a Swedish cohort [46] have recently shown that the most common infections in the year following SLE diagnosis, were influenza, herpes zoster, pneumonia and urinary tract infections, and that doses of oral GCs <5 mg/day were associated with a lower risk of suffering such infections. Indeed, Abe et al.^47^ have suggested that the use not only of doses >7.5 mg/day, but also of doses of 5.0 to 7.5 mg/day of prednisolone or equivalent, may pose an increased risk for infection in SLE patients.

### Cardiovascular disease

It is well known that the incidence of cardiovascular events is increased in SLE, particularly, in patients with LN and chronic kidney disease.^48^ Although the activity of lupus also plays an important role, it is the long-term cumulative dose of GCs that is crucial in developing atherosclerosis. The use of medium-to high-dose GCs has been associated with increased cardiovascular events, subclinical atherosclerosis and coronary disease.^49,50^ The risk of both diabetes and hypertension has been associated with the duration of exposure to GCs.^51,52^ The Systemic Lupus International Collaborating Clinics (SLICC) Registry for Atherosclerosis inception cohort, recruiting 1686 SLE patients at early stages of disease course, has shown that metabolic syndrome was associated with higher disease activity, history of LN and higher oral doses of GCs.^53^

### Bone disease

GCs increase bone resorption and decrease bone formation. Avascular osteonecrosis and osteoporotic fractures have been associated with exposure to prednisone.^5^ The prevalence of osteoporosis in SLE is 10 to 20%, with up to 20% of patients experiencing vertebral fractures. Osteoporosis has been associated with higher cumulative doses, and prolonged administration of GCs.^54,55^ Osteonecrosis has been reported in 5–15% of patients with SLE and has been associated with high cumulative doses of GC during the first months of treatment.^54,56–58^ Therefore, every effort should be made to reduce GC burden and maintain bone health, especially in post-menopausal women.

## HOW TO MAXIMISE CLINICAL EFFICACY AND MINIMISE GC BURDEN

The effect of GCs on damage accrual and the comparable efficacy of low doses of prednisone in SLE activity has been consistently reproduced.^4 –7,16–18,20,21,36,59–61^ There are several tools that aim to improve clinical efficacy in lupus activity while reducing GC toxicity.

### Concomitant use of antimalarials

Hydroxychloroquine (HCQ) is the background therapy for all SLE patients and should be maintained long-term unless maculopathy is confirmed.^1^ HCQ has shown a main protective role in achieving remission and preventing SLE flares as reported in the GLADEL^62^ and SLICC63 cohorts. Moreover, the longer duration of HCQ therapy is associated with prolonged clinical remission.^17,64,65^

Beyond controlling SLE activity, HCQ has a major role in damage prevention, one of the main targets of treatment.^1^ Patients treated with HCQ have shown to prevent damage in the LUMINA,^66^ SLICC,^67^ and LuLa^68^ cohorts. The use of HCQ has also been found one of the most important protective factors of renal damage, as shown in the LUMINA^69^ and the GLADEL70 cohorts. Finally, a study from the Lupus-Cruces cohort showed the protection from cardiovascular damage by longer use of HCQ among patients with SLE and antiphospholipid antibodies.^71^ As an expected result, HCQ has been shown to increase the survival of lupus patients in cohorts across the world. ^72–74^

### Intravenous GCs pulses

By means of non-genomic mechanisms, MP result in a rapid and potent anti-inflammatory effect, also priming the immune cells for the upcoming genomic effects.^26,75^ Furthermore, a recent work has investigated the role of MP therapy in the differentiation of regulatory T cells (Tregs).^76^ By inducing the apoptosis of CD4+ T cells and, subsequently, the production of transforming growth factor β (TGFβ), MP can promote an increase of circulating Tregs. These findings support the contribution of MP to immunomodulation and provide a possible explanation for the longer prolonged remission observed in patients treated with MP, compared to those who did not received MP in the induction phase, despite the reduction of oral GCs.^17,21^

Intravenous pulse GC therapy is often reserved for patients with more severe disease.^1,8,9^ However, moderate, and even non-rapidly-responding mild flares would also benefit from MP, both in terms of efficacy and of lower starting doses and faster tapering of oral GCs.^5,16,21,59–61^ Observational studies from the Lupus Cruces-Bordeaux inception cohorts support the more rapid achievement of prolonged remission and the reduction of long-term damage using schedules that combine MP with reduced prednisone doses, both in LN^18,21^ and in other clinical scenarios.^17^

Although the use of MP has been associated with higher risk of infection in some cohort studies,^77^ these studies did not control for MP dose. Moreover, a recent retrospective study has reported a protective role for herpes zoster of MP during induction therapy in LN patients.^78^ Although most guidelines have traditionally suggested MP at high doses up to 1000 mg/day for 3 days for severe lupus flares,^1,8,9,12^ the pharmacodynamics of GCs and the clinical evidence of no additional benefit from higher doses makes MP 250–500 mg/day enough to treat severe lupus flares, with both high efficacy and fewer associated serious infections.^33,79^ Besides, MP 125–250 mg/day seems appropriate for treating mild to moderate flares, as well as for the combination with each dose of cyclophosphamide during the induction therapy of severe manifestations.^18^ Although a recent study has suggested that cumulative doses of MP over 3 g would be related to increased damage, this study is severely limited by the lack of adjustment for SLE severity and oral GC burden.^80^

### Prompt introduction of immunosuppressive drugs

The efficacy of schemes combining MP, oral GCs and potent immunosuppressive drugs has been consistently demonstrated in LN. Observational studies of the Lupus Cruces-Bordeaux induction cohorts have shown that 86% of patients treated with cyclophosphamide plus MP in the induction phase achieved complete renal remission at a 0.5 protein/creatinine ratio at 12 months,^18^ a higher proportion than among those receiving cyclophosphamide alone or mycophenolate (56% and 47%, respectively). In addition, the cyclophosphamide-MP group received lower doses of prednisone within 6 months (mean 8.5 mg/d, vs. 15 mg/d in the mycophenolate group vs. 24 mg/d in the cyclophosphamide group, respectively). Ongoing research is focused on the steroid sparing effects of biologics in remission induction and also on the role of early initiation of biologic drugs as first line treatments,^81^ which has been included in recent guidelines.^1,14^ However, it should be noted that the reduction of GC doses is possible in most lupus patients by using old, cheap and widely available immunosuppressive and antimalarial drugs, as has been shown in the Lupus-Cruces cohort.^16–18^

### Fixed-rate GC tapering scheme

Most recommendations set a target maintenance dose of prednisone ≤5–7.5 mg/day without providing a specific tapering scheme. The EULAR/ERA/EDTA guidelines for LN [12] proposed doses of prednisone 0.3–0.5 mg/kg/day for up to 4 weeks, tapered to ≤7.5 mg/day by 3 to 6 months. The most recent KDIGO guidelines^14^ propose a reduced-dose scheme from 40 mg/day to 5 mg/day in 10 weeks, based on the AURA clinical trial protocol.^61^ In a similar scenario, the Lupus-Cruces LN protocol includes the fixed tapering from 20–30 mg/day to 5 mg/day of prednisone in 12 weeks, combined with intravenous cyclophosphamide plus MP every two weeks.^18,20,21^

Therefore, a fixed-rate GC tapering scheme is possible, and also a valuable tool to achieve a gradual reduction in GC burden, independent of clinical evolution. Persistent disease activity during GC tapering should lead to the intensification of immunosuppressive/biologic treatment and the repeated use of MP for rapid effects free from the genomic-mediated toxicity, not to slowing down GC tapering.

## IS IT POSSIBLE AND SAFE TO WITHDRAW GCs?

Rapid GC discontinuation has been included in the strategy of minimising glucocorticoid exposure.^1^ Whether stopping GCs is achievable and who could benefit from withdrawal minimising the risk of disease flares is still a matter of debate. All guidelines agree in considering withdrawal of GCs after prolonged remission on stable treatment.^1,9,11–14^ (**Table 1**). Of note, none of them provide a specific tapering scheme until definitive discontinuation. The recent EULAR/ERA/EDTA^12^ and EULAR 2023 guidelines^1^ propose gradual withdrawal of treatment after at least 3 to 5 years of therapy in complete clinical/renal response. The British Society for Rheumatology^9^ prompts GC discontinuation after at least 3 years of remission. The 2024-KDIGO guidelines^14^ suggest GC discontinuation after complete renal response without extrarenal manifestations for about one year. It is remarkable that all guidelines agree in recommending that GC withdrawal should be attempted before stopping immunosuppressive drugs.

This issue has one of the central topics of academic debate since 2020, after the publication of the clinical trial conducted by Mathian et al.^82^, evaluated the withdrawal of prednisone from 5 mg/day to zero, after at least a year of quiescence disease on stable therapy. The authors concluded that withdrawing GC over continue them would result in a four-fold increase in the risk for lupus flare, with no increased damage accrual among those continuing on prednisone. A meta-analysis including Mathian’s clinical trial and 14 observational studies has shown that, globally and quite consistently across the different studies, around 1/4 patients would flare within the following months upon GC withdrawal, almost half of them suffering severe flares.^83^

A history of LN and serologic activity have been identified as risk factors of flare after stopping GCs; on the contrary, prolonged therapy with HCQ has been shown a protective factor.^84–87^ Of note, the use of immunosuppressive drugs has not decreased the risk for flares, even being associated with a higher rate of lupus reactivations in some studies.^84,85^

Prolonged remission before attempting withdrawal and a gradual discontinuation of prednisone over periods longer than 3 months do increase the chance of an uneventful course of the disease after stopping GCs.^86,88–89^ Li et al.^90^ have recently reported the results of an observational study of 365 patients with LN, of whom 5.8% achieved the successful discontinuation of GC after 5 years of complete remission on stable treatment. The average duration of GC reduction from 7.5 mg/day to zero lasted for approximately 25 months. However, less than 50% of the patients were treated with HCQ alone, and most of them continued immunosuppressive drugs after prednisone withdrawal. In the same line, Tani et al.^91^ reported that in their SLE cohort of 148 patients, 91 attempted GC withdrawing with a success rate of 84.5%. Failure to withdraw was related to disease activity and with shorter time from the last flare. Therefore, the key for success when withdrawing GC is a good selection of those patients with a long remission time and a slow but steady reduction of prednisone over a time span of months-to-years.^92,93^

It is worth noting that a small subgroup of patients may experience a worsening of general symptoms, such as sleep or emotional disturbances, unrelated to lupus activity, in the following weeks after definitive withdrawal of prednisone. These symptoms could be explained by a downregulation of the adrenal axis.^94^ Furthermore, up to 37% of patients will have some degree of adrenal insufficiency, which could persist for more than 3 years in 15% of them.^95^ Both scenarios would lead to a worsening of the quality of life and, therefore, the appropriate management, mostly resuming low dose prednisone or treating adrenal insufficiency would be necessary.^94,96^ A diagnostic algorithm for adrenal insufficiency in patients treated with GCs, according to the daily dose and time of exposure, have been proposed.^97^ However, symptoms of withdrawal are mild and limited to few weeks in the majority of patients.

Finally, although GC withdrawal has been associated with a reduction in the risk of damage accrual compared with GC continuation in some studies, this result may be greatly influenced by GC load over the years preceding the attempt.^98^ It is therefore much more important how long it takes to reduce the initial dose to 5 mg/day than how quickly GCs are withdrawn once this point is reached.^99^ Studies with longer follow-up periods after withdrawing GC are needed to assess whether GC related damage is increased or not in patients continuing on low doses long term.

## PRACTICAL APPROACH TO GCs USE IN SLE: THE CRUCES PROTOCOL

Based on the pharmacological and clinical evidence and the balance between benefits and risks, high-dose oral GCs must be always avoided at any time in the management of SLE. Mild lupus flares can be treated with minor increases in prednisone dose. For moderate to severe flares, MP have a more rapid onset of action, allowing the use of lower doses of prednisone, never higher than 30 mg/day, with a rapid taper to doses of 5-2.5 mg/day within a few weeks. Such weaning must be done regardless of the evolution of SLE, which in case of persisting activity should be treated by using early combinations of immunosuppressive and, if needed, biologic drugs, with repeated MP offering a bridging therapy until other therapies are fully effective. HCQ should always be maintained except in the very unusual event of well-confirmed toxicity. Our proposal for the treatment of SLE according to clinical scenarios and some tips for the correct use of GCs are shown in **Tables 2** and **3**.

**Table 2.** Proposal for the treatment of Systemic Lupus Erythematosus according to clinical scenarios.

**Long-term HCQ 200 mg/d with ophthalmological survey**					
**Clinical setting**	**Pulse therapy**	**Initial dose of prednisone**	Tapering scheme*	**Maintenance dose**	**Discontinuation scheme**
**Mild flares** (Polyarthralgia, small joint mono-oligoarthritis, limited skin lesions)	Not needed initially	≤7.5 mg/d		2.5–5 mg/d	Clinical remission for at least 3–5 years on prednisone ≤5 mg/d,
Moderate flares (Polyarthritis, moderate thrombocytopenia (20,000 50,000/mm3), haemolytic anaemia with a low rate of haemolysis, widespread skin lupus lesions, non-severe pericardial effusion/pericarditis, pleural effusion, **mild flares non responding to treatment**)	MP 125–250 mg/d for 3 days	Prednisone ≤10–15 mg/d	Reduced 1–2 weekly (10-7.5) to 5 mg/d		then Previous gradual withdrawal of immunosuppressive drugs, then
**Severe flares** (Lupus nephritis, pneumonitis, severe thrombocytopenia (<20,000/mm3), haemolyticanaemia with a high rate of haemolysis, severe pericardial effusion, refractory pleural effusion, severe neuropsychiatric manifestations, **moderate flares non responding to treatment**)	MP 250–500 mg/d for 3 days or dexamethasone 40 mg for 4 daysRepeat pulses if persistent activityMP 125 mg added to each fortnightly dose of iv cyclophosphamide, if used	Prednisone 20–30 mg/d	2–4 weekly reduced (20-15-10-7.5) to 5 mg/d (in 12 weeks)		Slow tapering (≤3–6 months) by 2.5 mg/d on alternate days until discontinuation

MP: methylprednisolone pulses; HCQ: hydroxychloroquine.

* If the clinical course does not allow a reduction of prednisone in moderate disease, additional therapy should be added depending on specific organ involvement: mepacrine and/or methotrexate in skin, articular, or serosal diseases, azathioprine in immune cytopenias and in women with forthcoming pregnancy plans, belimumab in refractory disease despite immunosuppressive therapy. In severe flares, potent immunosuppressive drugs should be added from the beginning: cyclophosphamide in renal, CNS, lung disease, mycophenolate +/- calcineurin inhibitors in renal disease, rituximab in life-threatening disease or in severe disease without rapid response to therapy.

**Table 3.** Do’s and Don’ts in the management of glucocorticoids in patients with Systemic Lupus Erythematosus.

**Do’s**

✓ Maintain long-term HCQ unless toxicity is confirmed.
✓ Adjust induction therapy to the severity of the disease.
✓ Restrict maintenance dose of prednisone to ≤5 mg/day (preferably ≤2.5 mg/day).
✓ Use MP (125–500 mg/day for 3 days) to treat moderate to severe flares.
✓ Use immunosuppressive drugs form the beginning to treat moderate to severe flares.
✓ Consider MP to treat mild flares that do not respond to prednisone up to 7.5–10 mg/day within one week.
✓ Use immunosuppressive drugs to treat mild flares that do not respond to antimalarials and prednisone up to 5 mg/day.
✓ Consider the discontinuation of prednisone after clinical remission for at least 3–5 years.
✓ Start prednisone withdrawal after immunosuppressive drugs discontinuation, and slowly taper for at least 3–6 months until definitive discontinuation.

**Don’ts**

✗ Ever start prednisone at doses higher than 30 mg/day.
✗ Ever use maintenance dose of prednisone >5 mg/day.
✗ Start biologics just as a steroid reduction strategy.
✗ Consider prednisone withdrawal before clinical remission has been achieved for at least 3–5 years.
✗ Taper prednisone to zero over periods shorter than 3 months.
✗ Stop HCQ unless toxicity is confirmed.

HCQ: hydroxychloroquine; MP: methylprednisolone pulses.

## CONCLUSIONS

GCs still constitute the best and more rapid way to treat acute inflammatory conditions, including lupus flares. However, medium-long term toxicity associated to their continuous use makes GCs a double-edge sword. Using MP, from 125 to 500 mg/d for short periods of time, usually 3 days, starting prednisone at doses ≤30 mg/d followed by a rapid and non-stop tapering to 5 mg/d over few weeks and never keeping maintenance doses over 2.5–5 mg/d is the best way to take advantage of their full anti-inflammatory effects while minimising the chance for GC-related side effects and damage.
